# Human genetic basis of severe or critical illness in COVID-19

**DOI:** 10.3389/fcimb.2022.963239

**Published:** 2022-09-20

**Authors:** Xiao-Shan Ji, Bin Chen, Bi Ze, Wen-Hao Zhou

**Affiliations:** ^1^ Department of Neonatology, Children’s Hospital of Fudan University, National Children’s Medical Center, Shanghai, China; ^2^ Key Laboratory of Birth Defects, Children’s Hospital of Fudan University, National Children’s Medical Center, Shanghai, China

**Keywords:** COVID-19, disease severity, critical illness, genetic, SARS-CoV-2

## Abstract

Coronavirus Disease 2019 (COVID-19) caused by the novel severe acute respiratory syndrome coronavirus 2 (SARS-CoV-2) has led to considerable morbidity and mortality worldwide. The clinical manifestation of COVID-19 ranges from asymptomatic or mild infection to severe or critical illness, such as respiratory failure, multi-organ dysfunction or even death. Large-scale genetic association studies have indicated that genetic variations affecting SARS-CoV-2 receptors (angiotensin-converting enzymes, transmembrane serine protease-2) and immune components (Interferons, Interleukins, Toll-like receptors and Human leukocyte antigen) are critical host determinants related to the severity of COVID-19. Genetic background, such as 3p21.31 and 9q34.2 loci were also identified to influence outcomes of COVID-19. In this review, we aimed to summarize the current literature focusing on human genetic factors that may contribute to the observed diversified severity of COVID-19. Enhanced understanding of host genetic factors and viral interactions of SARS-CoV-2 could provide scientific bases for personalized preventive measures and precision medicine strategies.

## 1 Introduction

**Figure 1:**
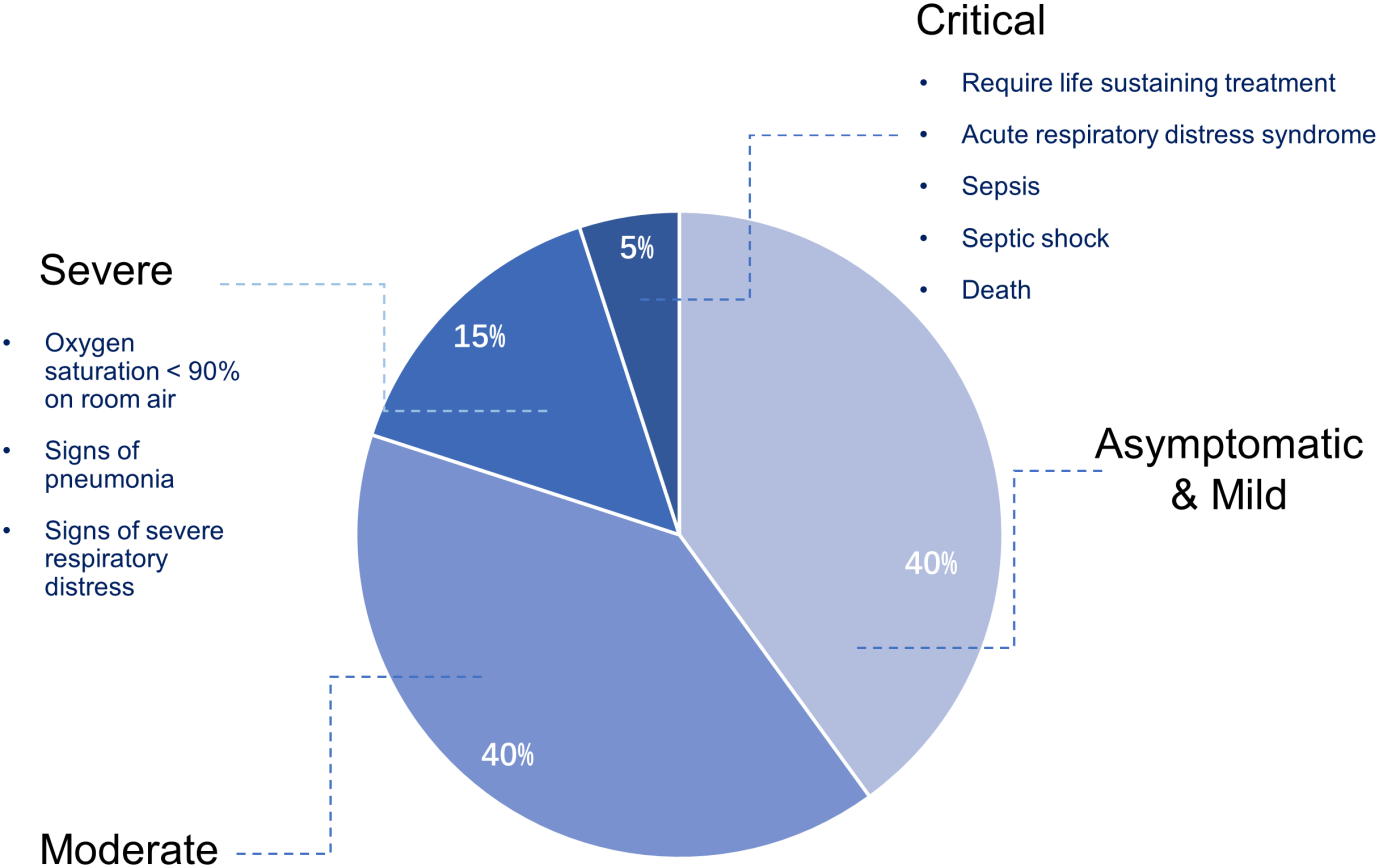
Overview of clinical characteristics of COVID-19.

Coronavirus Disease 2019 (COVID-19) was caused by the novel severe acute respiratory syndrome coronavirus 2 (SARS-CoV-2). Since the first case reported in December 2019, it has been spreading worldwide and was announced a global pandemic in March 2020 (Taylor, 2022). COVID-19 presents a wide spectrum of manifestations, ranging from asymptomatic infection to critical clinical course (**Figure 1**). Though most cases are now known to be asymptomatic or mild, approximately 15% of infected patients developed severe disease and 5% progressed to critical status, leading to deleterious acute respiratory distress syndrome (ARDS), multi-organ dysfunction and death (Baj et al., 2020; Zhou et al., 2020).

Several risk factors that could predict the severity of disease have been identified, including age, male gender, smoking, underlying comorbidities such as hypertension, diabetes mellitus, cardiac disease, chronic lung disease and cancer, clinically apparent immunodeficiencies, local immunodeficiencies and pregnancy (Williamson et al., 2020; Grasselli et al., 2021). Nevertheless, these conditions do not fully explain the variability in COVID-19 disease severity between individuals, and severe cases were observed in young individuals without pre-existing medical conditions, sometimes clustering in families, suggesting genetic background might be a risk factor (Yousefzadegan and Rezaei, 2020).

Several gene variants of infected patients were reported to explain the different levels of severity among individuals and their outcomes, which may provide a better understanding of host protein-SARS-CoV-2 interactions. Also, it sheds light on stratifying individuals according to risk, thus allowing for the prior protection of those at greater risk, and ideally, for innovative personalized treatments. To this end, we conduct a review on current studies focusing on associations between human genetic factors and the level of severity of COVID-19.

## 2 SARS-CoV-2 recognition and immune responses

**Figure 2:**
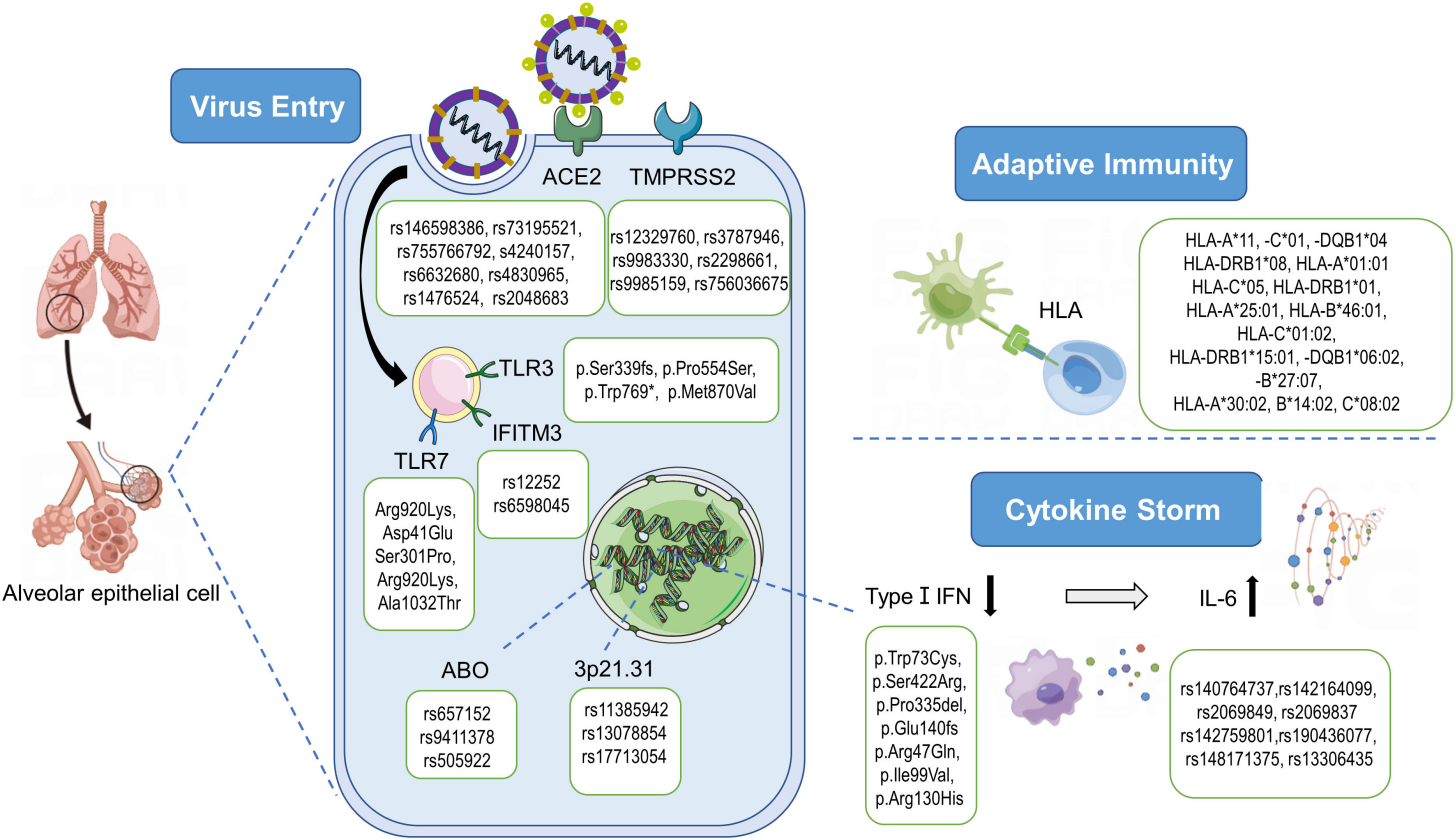
Pathogenesis of SARS-CoV-2 and genetic variants associated with severe COVID-19. After the recognition of ACE2 and the priming by TMPRSS2, SARS-CoV-2 enters the cell and starts the replication process. The innate immune response signaling cascade starts with the recognition of pathogen‐associated molecular patterns (PAMPs) by endosomal toll‐like receptors (TLRs). SARS-CoV-2 is able to inhibit the type I IFN responses in infected cells, leading to the cytokine storm characterized by an increase of inflammatory cytokines/chemokines such as IL-6. As an antiviral mechanism, antigen-presenting cells present antigenic peptides through the Major Histocompatibility Complex (MHC) class I and class II molecules to T cells. And 3p21.31 and ABO loci are significantly associated with Covid‐19 severity. ACE2, angiotensin‐converting enzyme‐2; TMPRSS2, transmembrane serine protease‐2; HLA, human leukocyte antigen; IFN, interferon; TLR, Toll-like receptor 7; IFITM3, interferon induced transmembrane protein 3; IL, Interleukin. By Figdraw (www.figdraw.com).

There are two distinct biological steps relevant to the severe presentation of COVID-19: viral recognition and immune responses (**Figure 2**). First, the spike protein (S) on SARS-CoV-2 binds to the host ACE2 (Angiotensin-2 Conversion Enzyme) receptor (Dong et al., 2020). Following the receptor binding, Transmembrane and Serine Protease 2 (TMPRSS2) will trigger a proteolytic cleavage of the S domains to mediate membrane fusion (Hoffmann et al., 2020). Paired basic amino acid-cleaving enzyme (Furin) can also catalyze S protein proteolytic cleavage.

After the virus entering a target cell, the innate immune response is initiated with the recognition of SARS-CoV-2 by pattern recognition receptors such as Toll-like receptors (TLRs) 3, 7, 8 and 9. The TLR3 response triggers the activation of NOD-Like Receptor family and Pyrin domain-containing 3 protein (NLRP3) inflammasome pathway, which induces caspase-1-dependent cleavage and secretion of key proinflammatory cytokines interleukin-1β (IL-1β) and IL-18 (Brodin, 2021), inducing inflammation and coagulopathy (Hosseini et al., 2020). In adaptive immunity, T cells recognize a bimolecular complex of an epitope bound to the Major Histocompatibility Complex (MHC) class I and II. CD4+ T cells play a critical antiviral role through promoting the secretion of pathogen-specific antibodies, whereas CD8+ T cells reduce the viral burden by killing the infected cells. It has been reported that T cells in critical patients seemed to be more active (Maamari et al., 2022). SARS-CoV-2 also triggers a robust B cell response, as IgM, IgG, IgA and neutralizing IgG antibodies can be detected in a few days after infection (Grifoni et al., 2021).

Commonly, the proinflammatory cytokines activate immune cells, notably monocytes and T lymphocytes, which clean the lung infection and help the patient recover. However, in serious cases, uncontrolled systemic hyper-inflammation, called “cytokine storm”, may occur (Kim et al., 2021). Though the pathogenesis of cytokine storm is not yet elucidated, two stages of the cytokine storm has been considered: the first stage is a temporary immune-deficient condition in which early responses of type I Interferon (IFN) are impaired; the secondary stage is an overactive immune state to compensate for the failure of target clearance (Blanco-Melo et al., 2020; McGonagle et al., 2020). A key feature of SARS-CoV-2 is its capability to shut down hosts’ IFN production, leading to a delayed or even overall suppressed type I IFN response (Zhang et al., 2021). An immune analysis performed in critical COVID-19 patients showed a low level of IFN activity and downregulation of IFN stimulated genes. On the contrary, cytokine and chemokine-related genes such as IL-6 and TNF-α were found to be increasingly expressed (Hadjadj et al., 2020). Taken together, hyper-inflammatory responses followed by impaired IFN signaling pathway are likely to determine the severity of COVID-19.

## 3 Genetic variations associated with severity of COVID-19

Considering the pathogenesis of COVID-19, the gene variants described for disease severity were classified whether they were related to host entry mechanism, immune system or other genes associated with severity of COVID-19. Gene variants that show protective or risk factors on the severity of COVID-19 are summarized in **Table 1**.

**Table 1:** Summary of genetic variations associated with COVID-19 severity.

Location	Gene(s)	Polymorphism(s)		Population	Ref.
		Risk	Protective		
Xp22.2	ACE2	rs146598386, rs73195521, rs755766792		Russian	(Shikov et al., 2020)
		s4240157, rs6632680, rs4830965, rs1476524, rs2048683		Caucasian	(Wooster et al., 2020)
		rs4830984		Worldwide	(Yang et al., 2022)
		rs2285666		Iranian	(Khalilzadeh et al., 2022)
			rs190509934	American	(Horowitz et al., 2022)
21p22.3	TMPRSS2	rs12329760 (p.Val160Met)		Italian	(Asselta et al., 2020)
		rs3787946, rs9983330, rs2298661, rs9985159	rs12329760 (p.V197M)	European	(Andolfo et al., 2021)
		rs756036675		Spanish	(Villapalos-Garcia et al., 2022)
			rs12329760 (p.V197M), rs2298659 (p.G296G)	Italian	(Monticelli et al., 2021)
		rs17854725, rs12329760, and rs4303795		Iranian	(Rokni et al., 2022)
21q22.1	IFNAR1	p.Trp73Cys, p.Ser422Arg, p.Pro335del		Chinese, Italian	(Zhang et al., 2020a)
	IFNAR2	p.Glu140fs		Belgian	(Zhang et al., 2020a)
		rs2236757		European	(Pairo-Castineira et al., 2021)
		Tyr322Ter		Asian	(Smieszek et al., 2021a)
		p.Ser53Pro		Canadian	(Duncan et al., 2022)
11p5.5	IFITM3	rs12252		Chinese, Saudi Arabian	(Zhang et al., 2020c)
		rs6598045		worldwide	(Kim and Jeong, 2021)
		rs12252 and rs34481144		British	(Nikoloudis et al., 2020)
12q24.13	OAS1	p.Arg47Gln, p.Ile99Val and p.Arg130His		Serbian	(Klaassen et al., 2020)
			rs10774671	Peruvian, Esan	(Wickenhagen et al., 2021)
		rs10735079		European	(Pairo-Castineira et al., 2021)
		rs1131454, rs4766676		British	(Magusali et al., 2021)
7p15.3	IL6		rs140764737, rs142164099, rs2069849, rs142759801, rs190436077, rs148171375, rs13306435	Italian	(Strafella et al., 2020)
			rs2069837	Chinese	(Gong et al., 2022)
			rs1800796, rs1524107, rs2066992	Chinese	(Chen et al., 2021)
1q21.3	IL6R		rs2228144, rs2229237, rs2228145, rs28730735, rs143810642	Italian	(Strafella et al., 2020)
		rs2228145		American	(Smieszek et al., 2021b)
20q13.13	TMEM189-UBE2V1	rs6020298		Chinese	(Wang et al., 2020)
Xp22.2	TLR7	Arg920Lys, Asp41Glu		Italian	(Mantovani et al., 2022)
		c.2129_2132del; p.Gln710Argfs*18; c.2383G>T; p.Val795Phe		Dutch	(van der Made et al., 2020)
		Ser301Pro, Arg920Lys, Ala1032Thr		Italian	(Fallerini et al., 2021)
4q35.1	TLR3	p.Ser339fs, p.Pro554Ser, p.Trp769*, p.Met870Val		Italian, Spanish	(Zhang et al., 2020a)
6p21.3	HLA	HLA-A*11, -C*01, and -DQB1*04	HLA-A*32	Spanish	(Lorente et al., 2021)
		HLA-DRB1*08		Italian	(Amoroso et al., 2021)
		HLA-C*05		74 countries	(Sakuraba et al., 2020)
		HLA-A*01:01g, HLA-B*08:01g and HLA-DRB1*03:01g	HLA-B*18:01, HLA-C*07:01 and HLA-DRB1*11:04	Italian	(Pisanti et al., 2020)
		HLA-A*25:01, HLA-B*46:01, and HLA-C*01:02	HLA-B*15:03	American	(Nguyen et al., 2020)
		HLA-A*11:01, -B*51:01, and -C*14:02		Chinese	(Wang et al., 2020)
		HLA-DRB1*15:01, -DQB1*06:02, and -B*27:07		Italian	(Novelli et al., 2020)
		HLA-A*30:02, B*14:02 and C*08:02	HLA-A*02:05-B*58:01-DRB1*08:01 and HLA-A*02:05-B*58:01-C*07:01	Sardinian	(Littera et al., 2020)
		HLA-A*01:01	HLA-A*02:01 and HLA-A*03:01	Russian	(Shkurnikov et al., 2021)
3p21.31	SLC6A20, LZTFL1, CCR9, FYCO1, CXCR6 and XCR1	rs11385942		Italian, Spanish	(Ellinghaus et al., 2020)
		rs13078854		American, British	(Shelton et al., 2021)
		rs17713054		British	(Downes et al., 2021)
9q34.2	ABO	rs657152		Italian, Spanish	(Ellinghaus et al., 2020)
		rs9411378		American, British	(Shelton et al., 2021)
12q24.33	GOLGA3	rs143359233		Chinese	(Wang et al., 2020)
19p13.3	DPP9	rs2109069		European	(Pairo-Castineira et al., 2021)
19p13.2	TYK2	rs11085727		European	(Pairo-Castineira et al., 2021)
2q24.2	IFIH1	rs1990760		Spanish	(Amado-Rodriguez et al., 2022)

*The separator used to separate gene names from alleles groups in the naming of HLA allele.

### 3.1 Genetic variations of human receptors

#### 3.1.1 ACE2

ACE2 is widely expressed in human tissues, especially in upper and lower respiratory tracts, heart, kidney, testis and gastrointestinal system (Bourgonje et al., 2020). Apart from the main receptor for SARS-CoV-2, ACE2 is also well-known for its downregulating the renin-angiotensin system (RAS), which is important for modulating the cardiovascular system (Bakhshandeh et al., 2021). However, the function of ACE2 is lost following the binding of virus, which may cause inflammation, thrombosis and death.

The expression level of ACE2 receptor, which differs among individuals across different ages, genders and ethnicities, potentially affects the severity of COVID-19. Based on the latest genome-wide association summary statistics for severe COVID-19, a recent study indicated an increased risk of severe COVID-19 for individuals who had genetically raised levels of circulating ACE2 protein (Yang et al., 2022). They also found that the variant rs4830984 was nominally significantly associated with severe COVID-19. A retrospective examination of nasal epithelium among people of different ages showed that expression level of *ACE2* gene was low in younger children but increased with age (Bunyavanich et al., 2020), which may explain why children have fewer and less severe symptoms compared with adults (Patel and Verma, 2020). In addition, compared with women, men are 65% more likely to develop severe complications or even die from COVID-19. This gender difference could be explained by the well-established role of androgen receptor signaling in modulating *ACE2* transcription, as well as its location on X chromosome (Samuel et al., 2020). Also, the *ACE2* gene expression varies among ethnic populations. A recent study based on expression quantitative trait locus (eQTL) found that people from an Arab background had lower levels of ACE2 compared with Europeans, possibly led to lower mortality in this population (Al-Mulla et al., 2020).

Genetic variations in *ACE2* may affect its binding with SARS-CoV-2 and the subsequent infection severity. Several missense changes, such as p.(Asn720Asp), p.(Lys26Arg), and p.(Gly211Arg), can affect the protein structure and stabilization, and therefore influence the internalization process of the virus (Benetti et al., 2020). Some other variants, such as rs961360700, are known to cause an increase in affinity for S protein (MacGowan et al., 2022; Ren et al., 2022). Accumulating evidence suggests that polymorphisms in *ACE2* gene may modulate inflammatory responses and thus may aggravate pulmonary and systemic injuries (Li et al., 2020). In a cohort of Russian COVID-19 patients, several rare *ACE2* variants (including rs146598386, rs73195521, and rs755766792) tended to cause an active inflammatory response to infection, which partially explained the variation of disease severity (Shikov et al., 2020). In another study, six variants (re4240157, rs6632680, rs1548474, rs4820965, rs1476524 and rs2048683) out of 61 evaluated ones were identified to be markedly associated with hospitalization (Wooster et al., 2020). A recent genome-wide association study (GWAS) identified a rare variant, rs190509934, that downregulated *ACE2* expression and reduced disease severity among COVID-19 patients (Horowitz et al., 2022).

Nevertheless, the relationship between *ACE2* polymorphism and COVID-19 severity remain controversial. A negative correlation between ACE2 expression and COVID-19 fatality at both population and molecular levels was reported (Chen et al., 2020; El Baba and Herbein, 2020). In addition, *ACE2* genetic variants were analyzed by whole-exome sequencing (WES) in 137 DNA samples of COVID-19 patients, compared with the 536 age-matched controls. They found that *ACE2* polymorphism was not associated with an increased risk of critical illness (Gomez et al., 2020). However, they indicated that the balance between ACE1 and ACE2 played a role in the severity of COVID-19. Another study also revealed a strong correlation between *ACE1* insertion/deletion (I/D) genotype with COVID-19 mortalities (Yamamoto et al., 2020). Larger cohort of severe/critical patients and further functional studies are required to reveal the role of *ACE2* genotypes in COVID-19.

#### 3.1.2 TMPRSS2

The *TMPRSS2* gene, located on the human chromosome 21q22.3, encodes a serine protease enzyme that primes the S protein of SARS-CoV-2, allowing fusion of viral and cellular membrane (Baughn et al., 2020). *TMPRSS2* is a key gene in prostate cancer and its transcription is regulated by androgen. Thus, TMPRSS2 expression and enzymatic activity was detected significantly higher in males than in females, which may explain the male predominance of higher severity and mortality (Alshahawey et al., 2020; Okwan-Duodu et al., 2021). Also, TMPRSS2 expression increases with aging in mice and humans, and this may relatively protect children from severe illness (Rossi et al., 2021; Schuler et al., 2021). The localization of the gene on 21q22.3 place Down syndrome individuals at high risk for critical illness (De Toma and Dierssen, 2021), and its oncogenic role may be related to poor outcomes of cancer patients with COVID-19 as well (Stopsack et al., 2020).

Seven variants (rs3787946, rs9983330, rs12329760, rs2298659, rs2298661, rs9985159 and rs756036675) within *TMPRSS2* were identified to be associated with severe COVID-19 (Andolfo et al., 2021; Monticelli et al., 2021; Villapalos-Garcia et al., 2022). Among them, rs12329760 (p.Val197Met) emerged as a common variant that weakened TMPRSS2 protein stability and inhibited the binding of S protein and ACE2 (Wang et al., 2020). It played a protective role and appeared less in critical patients than in mild and general cases (Wang et al., 2020; Monticelli et al., 2021; David et al., 2022). However, rs12329760 (p.Val160Met) and 2 distinct haplotypes trigger higher TMPRSS2 expression may explain the significantly higher severity and mortality rates in Italy than those in East Asia (Asselta et al., 2020). It was suggested that more genotyping studies of COVID-19 was needed to explore the contribution of *TMPRSS2* variants to clinical outcomes (Stopsack et al., 2020).

### 3.2 Genetic variations of immunity components

#### 3.2.1 Interferons

Interferons (IFNs) are a family of specialized cytokines central to antiviral immunity. Viral recognition induces IFN production, which in turn triggers the transcription of IFN-stimulated genes (ISGs), mediating antiviral responses (Yang et al., 2021). Specifically, type I IFNs are the first line of defense against viral infections, and IFN-I signaling is required for the recruitment of pro-inflammatory cells in the lung (Ramasamy and Subbian, 2021). It was reported that inborn errors of type I IFNs were the genetic and immunological basis of at least 15% of cases of critical COVID-19 pneumonia (Hadjadj et al., 2020).

IFN-I signaling is initiated by the binding of IFN-I to the interferon receptor (IFNAR) complex, composed of IFNAR1 and IFNAR2 at the same proximal location (Schreiber, 2020). *IFNAR1* (p.Trp73Cys, p.Ser422Arg, p.Pro335del) and *IFNAR2* (p.Glu140fs) variants were identified in patients with life-threatening COVID-19, highlighting the importance of type I IFN production in severe disease (Zhang et al., 2020a). A GWAS also reported that an intron variant rs2236757 in the *IFNAR2* gene increased the odds of severe COVID-19 (Pairo-Castineira et al., 2021). Loss-of-function mutations in *IFNAR2* including Tyr322Ter may increase susceptibility to critical COVID-19 infection, especially Asian descent populations, where this variant is more prevalent (Smieszek et al., 2021a).

The interferon-induced transmembrane proteins (IFITM) are a group of proteins localized in the plasma and endolysosomal membranes, preventing viruses from traversing the cellular lipid bilayer (Shaath et al., 2020). Homozygosity for the C allele of rs12252 within the *IFITM3* gene was associated with the severity of COVID-19 (Zhang et al., 2020c). Rs34481144, another polymorphism of *IFITM3*, was reported to be associated with increased severity in influenza. It has been reported that the combined haplotypes of rs12252 and rs34481144 implicated in more severe outcomes of COVID-19 (Nikoloudis et al., 2020). However, a meta-analysis indicated that rs34481144 was not correlated to COVID-19 severity (Li et al., 2022).

2′‐5′‐Oligoadenylate synthase (OAS) family genes are induced by IFNs at the early phase of viral infection. Once in the right place, OAS1 binds to dsRNA structures of the SARS-CoV-2, leading to the viral RNA degradation and inhibition of viral replication (Wickenhagen et al., 2021). A common polymorphism in *OAS1* (rs10774671), where the protective allele resulted in a more active *OAS1* enzyme, probably led to less severe COVID-19 (Wickenhagen et al., 2021). On the contrary, decreased expression levels of *OAS1* was implicated in COVID-19 disease severity (D'Antonio et al., 2021). Three variants (p.Arg47Gln, p.Ile99Val and p.Arg130His) were detected to impair OAS1 activity and weaken its bond with RNA (Klaassen et al., 2020). Also, a recent GWAS suggested that the variant rs10735079 was associated with critical illnesses in COVID-19 (Pairo-Castineira et al., 2021). In addition, *OAS1* was identified as a putative new risk gene for Alzheimer’s disease, and 4 alleles within *OAS1* gene were identified to contribute to both the high incidence of Alzheimer’s disease and critical illness of COVID-19 (Magusali et al., 2021).

#### 3.2.2 Interleukin

As mentioned above, cytokine storm plays a critical role in severe COVID-19 cases, in which increased levels of cytokines are observed in plasma blood. Interleukin 6 (IL-6) is a soluble mediator in response to infections and tissue injuries (Tanaka et al., 2014). In COVID-19, critically ill patients showed significantly higher levels of IL-6, indicating that IL-6 was a strong predictor for disease severity and survival possibility (Zhang et al., 2020b). The association of *IL-6* polymorphisms with cytokine expression and disease severity have been reported. Seven variants in *IL-6* (rs140764737, rs142164099, rs2069849, rs142759801, rs190436077, rs148171375, rs13306435) and five variants in *IL-6R* (rs2228144, rs2229237, rs2228145, rs28730735, rs143810642) appeared to alter the binding of IL-6 and IL-6R, which can be implicated in the pathogenetic mechanisms associated with COVID-19 severity and its complications (Strafella et al., 2020). A recent GWAS found that the genetic variant rs2069837 in *IL-6* decreased the expression of IL-6 in the serum and was protective against critical COVID-19 (Gong et al., 2022). An Asian-common *IL-6* haplotype defined by promoter SNP rs1800796 and intronic SNPs rs1524107 and rs2066992 was detected to be associated with a lower risk of severe symptoms. Mechanistically, the protective allele disrupted the CTCF-binding locus at the *IL-6* intron and resulted in attenuated IL-6 induction in response to viral infection (Chen et al., 2021). On the contrary, the minor allele rs2228145 was associated with higher plasma IL-6 levels in severe COVID-19 patients (Smieszek et al., 2021b).

Beyond IL-6, IL-1 is also a highly active proinflammatory cytokine. A Chinese cohort investigated 22.2 million genetic variants among 332 COVID-19 patients, rs6020298 within *TMEM189-UBE2V1*, a component of IL-1 signaling pathway, was found to be the most significant SNP associated with severity (Wang et al., 2020).

#### 3.2.3 Toll‐like receptors

TLRs are a family comprised of 11 transmembrane proteins, which are crucial components in the initiation of innate immune responses (Szeto et al., 2021). TLRs recognize pathogen-associated molecular patterns and trigger the production of pro-inflammatory cytokines as well as type I and II interferons system. TLR3 is the most widely expressed TLR that binds to double‐stranded RNA viruses, while TLR7 and TLR8 recognize single‐stranded RNA viruses (Mantovani et al., 2022). Inborn errors of TLR3-dependent type I IFN immunity have been found in life‐threatening COVID‐19 patients, and eight genetic loci have been identified (Zhang et al., 2020a). The polymorphism L412 in *TLR3* inhibited autophagy and made males at risk of severe COVID-19 (Croci et al., 2021). X-linked TLR7 deficiency has been identified as a novel immunodeficiency with an increased susceptibility to severe or critical COVID-19 infection and TLR7 has been established as a critical mediator of IFN-I immunity against the virus (Solanich et al., 2021). The burden of rare variants in *TLR7* was found to be significantly higher in patients with severe COVID-19 in pan-ancestry WES data from the UK biobank (Kosmicki et al., 2021). A recent study identified loss‐of‐function variants of *TLR7* (c.2129_2132del; p.Gln710Argfs*18; c.2383G>T; p.Val795Phe) in four severely affected young men from two unrelated families and among them found a lower production of IFNα and IFNγ proteins following stimulation (van der Made et al., 2020). Moreover, a nested case–control study identified *TLR7* loss-of-function variants in 2.1% of severely affected males but in none of the asymptomatic participants (Fallerini et al., 2021).

Since the production of IFN is mediated *via* the TLR7 signaling pathway, therapies that directly stimulate endogenous TLR7 could have potential therapeutic benefit for the prevention and treatment of severe COVID-19 infection (Szeto et al., 2021). In addition, genetic variations in *TLR7* that located on the X chromosome, may be a possible explanation of the sex biases in COVID-19 severity. Among women, *TLR7* may escape X-inactivation, leading to higher basal expression levels and elevated downstream IFN responses (van der Made et al., 2020).

#### 3.2.4 Human leukocyte antigen

The Human Leukocyte Antigen (HLA) system, containing nearly 27,000 alleles in three distinct classes of genes (Class I, II and III), is the most highly polymorphic region in the human genome. HLA Classes I and II present antigenic peptides to T lymphocytes and enable the immune system to discriminate between self and foreign proteins (Lorente et al., 2021). In patients of COVID-19, different adaptive immune responses have been observed according to disease severity, including distinct IgM levels and S protein IgG titers (Ovsyannikova et al., 2020). As HLA plays a critical role in antigen presentation, different polymorphisms may potentially alter the severity of the disease.

Specific risk and protective *HLA* alleles for COVID-19 severity and mortality have been detected in several studies. A study evaluated the HLA binding affinity of all possible 8-mers to 12-mers from the SARS-CoV-2 proteome and noted three peptide-presenters (HLA-A*25:01, B*46:01, and C*01:02) that were most likely associated with severe infection (Nguyen et al., 2020). Another peptide binding prediction analyses showed that HLA-DRB1*08 alleles were unable to bind any of the viral peptides with high affinity, thus individuals with those alleles were at high risk of severe COVID-19 (Amoroso et al., 2021). Several studies concluded that HLA-A*11:01, HLA-B*51:01, HLA-C*14:02, HLA-DQB1*06:02 and HLA-B*27:0 were correlated with a higher COVID-19 mortality (Novelli et al., 2020; Wang et al., 2020; Shkurnikov et al., 2021). In contrast, HLA-A*02:01, HLA-A*03:01, HLA-B*18:01, HLA-C*07:01 and HLA-DRB1*11:04 showed an inverse relationship to the number of deaths (Novelli et al., 2020; Wang et al., 2020; Shkurnikov et al., 2021). HLA-A*11 was detected to predispose worse outcome of COVID-19 patients (Lorente et al., 2021), while another study suggested that HLA-A*11:01 could generate efficient antiviral responses (Tomita et al., 2020).

Considering the high gene density of *HLA* locus, it was suggested that complete HLA genotypes for each individual, rather than most frequent alleles, should be analyzed (Deng et al., 2021). Based on the allele frequency data of *HLA* in 74 countries, HLA-C*05 was identified as the most influential allele in increasing the mortality of COVID-19. Its receptor KIR2DS4fl is expressed on natural killer (NK) cells and recognizes viral peptides bound to HLA-C*05. It was hypothesized that this HLA-KIR pair induced immune hyperactivation and caused poor outcome (Sakuraba et al., 2020). An Italian study found that haplotype HLAA*01:01, HLA-B*08:01 and HLA-DRB1*03:01 contributed to the higher COVID-19 mortality in northern Italy. In contrast, HLA-B*18:01, HLA-C*07:01 and HLA-DRB1*11:04 directly correlated with the lower mortality in southern Italy (Pisanti et al., 2020).

Nevertheless, a study based on data from 6,919 infected individuals found that HLA genotypes as well as viral T-cell epitopes were not correlated with COVID-19 severity (Schetelig et al., 2021). More uniformly designed studies with the inclusion of global data are needed to clarify the role of single *HLA* alleles in COVID-19 severity. Furthermore, as COVID-19 may have variable potential epitopes with HLA complex, predicting good binds across *HLA* alleles may contribute to the design of an efficacious vaccine against COVID-19 (Prachar et al., 2020).

### 3.3 Other genetic variations

Apart from genes relevant to immune and SARS-CoV-2 receptors, other genetic variations have also been identified related to the severity of COVID-19. The association of loci 3p21.31 and 9q34.2 with COVID-19 severity were identified in two independent GWAS. The first study conducted in Italy and Spain revealed that rs11385942 at locus 3p21.31 and rs657152 at locus 9q34.2 were significantly associated with severe COVID‐19 with respiratory failure (Ellinghaus et al., 2020). And the second study found that rs13078854 at locus 3p21.31 and rs9411378 at locus 9q34.2 were risk alleles for severe COVID-19 phenotypes (Shelton et al., 2021). At locus 3p21.31, the association signal compromised 6 genes (*SLC6A20, LZTFL1, CCR9, FYCO1, CXCR6* and *XCR1*). Among them, *SLC6A20* encodes a transporter that functionally interacts with ACE2 receptor. *CXCR6* and *CCR9* encode chemokine receptors that are implicated in T cell differentiation and recruitment. *LZTFL1* encodes a cytosolic leucine-zipper protein widely expressed in pulmonary epithelial cells and regulates epithelial-mesenchymal transition (EMT), a viral response pathway (Downes et al., 2021). Recent studies found that rs35081325 and rs1024611 in *LZTFL1*, appeared to strongly associated with increased infection severity (Roberts et al., 2022; Ruter et al., 2022). And a study integrating expression quantitative trait locus (eQTL) mapping identified *SLC6A20* and *CXCR6* as causal genes that modulate COVID-19 risk (Kasela et al., 2021). However, another study identified *CCR9* and *SLC6A20* as potential target genes (Yao et al., 2021). As they all have a potentially relevant role in the pathophysiology of COVID-19, further studies will be needed to delineate effector genes at the 3p21.31 locus.

The association signal at locus 9q34.2 coincided with *ABO* locus, suggesting the role of ABO blood type in COVID-19 severity. It has been reported that A-group was a significant risk factor for developing a severe form of COVID-19, while O-group was protective against severe COVID19 illness or death (Gomez et al., 2021; Khasayesi et al., 2021). A recent replication analysis of reported COVID-19 genetic associations with eight phenotypes found that the lead *ABO* SNP, rs505922, replicated in all four susceptibility phenotypes and one severity phenotype (Roberts et al., 2022). It is still unclear how ABO blood types affect outcomes of COVID-19. A proteomic profiling analysis showed that the ABO locus mediated the risk by modulating CD209/DC-SIGN, a binding site for SARS-CoV-2 (Katz et al., 2020). Another study hypothesized that ABO blood group influenced the risk of venous thromboembolism, which is frequent in severe cases, by modifying glycosyltransferase activity (Ibrahim-Kosta et al., 2020).

*ApoE* is one of the highly co-expressed genes in type II alveolar cells in the lungs, and the *ApoE* e4e4 homozygous genotype was reported to increase the risk of severe COVID-19 (Kuo et al., 2020; Kurki et al., 2021). This may be explained by a regulatory mechanism underlying SARS-CoV-2 infection through ApoE interactions with ACE2 (Zhang et al., 2022).

Pedigree analysis in a Chinese family suggested that loss-of-function variants in *GOLGA3* and *DPP7* implicated in critically ill and asymptomatic COVID-19 patients as a monogenic factor (Wang et al., 2020). A GWAS performed in 2,244 critically ill patients with COVID-19 found significant associations in *DPP9, CCR2* and *TYK2*, all of which could cause inflammatory lung injury (Pairo-Castineira et al., 2021). A recent study found that patients with the TT variant in the *IFIH1* had an attenuated inflammatory response to severe SARS-CoV-2 infection, leading to better outcomes (Amado-Rodriguez et al., 2022).

## 4 Conclusions and perspectives

In this review, we provided an overview of genetic variants associated with COVID-19 severity. The variants influence at least two distinct biological progress: viral entrance to host cells and development of harmful inflammation. The world is still suffering from the COVID-19 outbreak, with high fatality rate in severe and critical patients. Therefore, identifying genetic markers associated with clinical outcomes of COVID-19 is helpful for classifying and safeguarding individuals at high risk, as well as finding potential therapeutic targets.

Future genetic studies need further sharing of individual-level data, yet ethical considerations such as perfecting genetic information-related legislation should also be considered. Furthermore, large-scale systematic investigations of the functional polymorphisms of these genes combining data among different populations would pave the way for personalized preventive measures and precision medicine strategies.

## Author contributions

All the authors listed, have made substantial, direct and intellectual contribution to the work, and approved it for publication. All authors contributed to the article and approved the submitted version.

## Funding

This study was funded by the National Key Research and Development Program of China (Nos. 2021YFC2701800).

## Conflict of interest

The authors declare that the research was conducted in the absence of any commercial or financial relationships that could be construed as a potential conflict of interest.

## Publisher’s note

All claims expressed in this article are solely those of the authors and do not necessarily represent those of their affiliated organizations, or those of the publisher, the editors and the reviewers. Any product that may be evaluated in this article, or claim that may be made by its manufacturer, is not guaranteed or endorsed by the publisher.
